# Photoelectrochemical and crystalline properties of a GaN photoelectrode loaded with α-Fe_2_O_3_ as cocatalyst

**DOI:** 10.1038/s41598-020-69419-8

**Published:** 2020-07-28

**Authors:** Martin Velazquez-Rizo, Daisuke Iida, Kazuhiro Ohkawa

**Affiliations:** Computer, Electrical and Mathematical Sciences and Engineering (CEMSE) Division, King Abdullah University of Science and Technology (KAUST), Thuwal, 23955-6900 Saudi Arabia

**Keywords:** Photocatalysis, Hydrogen energy

## Abstract

Nitrides are of particular interest in energy applications given their suitability to photocatalytically generate H_2_ from aqueous solutions. However, one of the drawbacks of nitrides is the decomposition they suffer when used in photoelectrochemical cells. Here, we report the improvement of the catalytic performance and chemical stability of a GaN electrode when it is decorated with Fe_2_O_3_ particles compared with an undecorated electrode. Our results show a higher reaction rate in the Fe_2_O_3_/GaN electrode, and that photocorrosion marks take more than 20 times longer to appear on it. We also characterized the crystalline properties of the Fe_2_O_3_ particles with transmission electron microscopy. The results show that the Fe_2_O_3_ particles keep an epitaxial relationship with GaN that follows the Fe_2_O_3_$\{{0003}\}||$GaN$\{{0001}\}$ and Fe_2_O_3_$[{11{\bar{ 2}}0}]||$GaN$[{1{\bar{ 1}}00}]$ symmetry constraints. We also characterized an Fe_2_O_3_ (thin film)/GaN electrode, however it did not present any catalytic improvement compared with a bare GaN electrode. The epitaxial relationship found between the Fe_2_O_3_ thin film and GaN exhibited the Fe_2_O_3_$\{{11{\bar{ 2}}0}\}||$GaN$\{{0002}\}$ and Fe_2_O_3_$[ {3{\bar{ 3}}00} ]||$GaN$[{11{\bar{ 2}}0}]$ symmetry constraints.

## Introduction

The increasing energy demand of humankind and the negative environmental impact of the consumption of fossil fuels are driving the development of alternative energy sources. One of the candidates to substitute fossil fuels is H_2_, which can generate energy through its direct combustion or in fuel cells without creating pollution. However, the non-pollutant attributes of H_2_ have been overshadowed by the lack of an environmentally friendly method for its manufacture.

One alternative to sustainably generate H_2_ is the photocatalytic water splitting using semiconductors^1^. Nitrides have received considerable attention in this field because, as a result of their band gap tunability^2–8^, they can absorb most of the energy of the solar spectrum. After the first demonstration of photoelectrochemical (PEC) H_2_ generation using GaN thin films as photoelectrodes^9^, and their subsequent improvement to perform chemical reactions without any external electrical bias^10^, the discovery of the NiO cocatalyst technology^11^ greatly boosted the expectations of nitride-based photocatalysts. NiO cocatalyst improved not only the Energy Conversion Efficiency (ECE) of nitride photoelectrodes, but also their lifetime, which has been demonstrated to be at least 500 h^12^. It has also been confirmed that NiO/nitride photoelectrodes can be used to generate more complex byproducts than H_2_, such as formic acid and methane from CO_2_ in artificial photosynthesis reactions^13–16^.

The improvement of the catalytic activity of GaN photoanodes by using metals or metal oxides as cocatalysts can be attributed to the introduction of active sites by the metal or oxygen atoms. For example, in water splitting on metal oxides, the Oxygen Evolution Reaction (OER) mechanism has been described conventionally as a four proton–electron transfer reaction in the metal-sites^17,18^. The OER activity of this mechanism is controlled by tuning the oxygen affinity of the adsorbed reaction intermediates. There is also evidence of another catalytic route where the lattice-oxygen participates in the reaction via the reversible formation of a superficial oxygen vacancy^19,20^. This mechanism shows that not only the electronic properties of the metal oxide surface are important but also its bulk electronic structure. The ECE of nitride-based photocatalysts can be improved if the role of their cocatalysts is understood. Finding new cocatalysts and analyzing the features they share with known cocatalyst materials, such as NiO, Ag^21^, Pt^22^, Rh_2-y_Cr_y_O_3_^23^ and CoO_x_^24,25^ is a crucial step towards the comprehension of their role in photocatalytic reactions. Moreover, it is preferable to find cocatalysts made of non-noble metals because of their availability and cost.

Hematite (α-Fe_2_O_3_, α is omitted henceforth) is a transition metal oxide that has been widely used and studied as photocatalyst and cocatalyst in water splitting and other chemical reactions, either alone or in combination with other materials^26–32^. However, there is little available information about its suitability to improve the photocatalytic properties of nitrides. In this work, we report the improvement of the catalytic activity in a GaN electrode decorated with Fe_2_O_3_ particles compared with a bare GaN electrode. Despite such improvement may be attributed to the introduction of active sites by Fe or O atoms in the Fe_2_O_3_, we propose a mechanism more consistent with our observations. The higher catalytic activity when the Fe_2_O_3_ is deposited as particles instead of as a thin film and the local formation of n^–^-n junctions suggest an enhancement due to the easier charge transport directly from GaN to the electrolyte. In addition, we present the characterization of the surface of the photoelectrode with Scanning Electron Microscopy (SEM) and the study of the crystalline properties of the cocatalyst with different Transmission Electron Microscopy (TEM) analytical techniques, including Electron Energy-Loss Spectroscopy (EELS).

## Results and discussion

**Figure 1:**
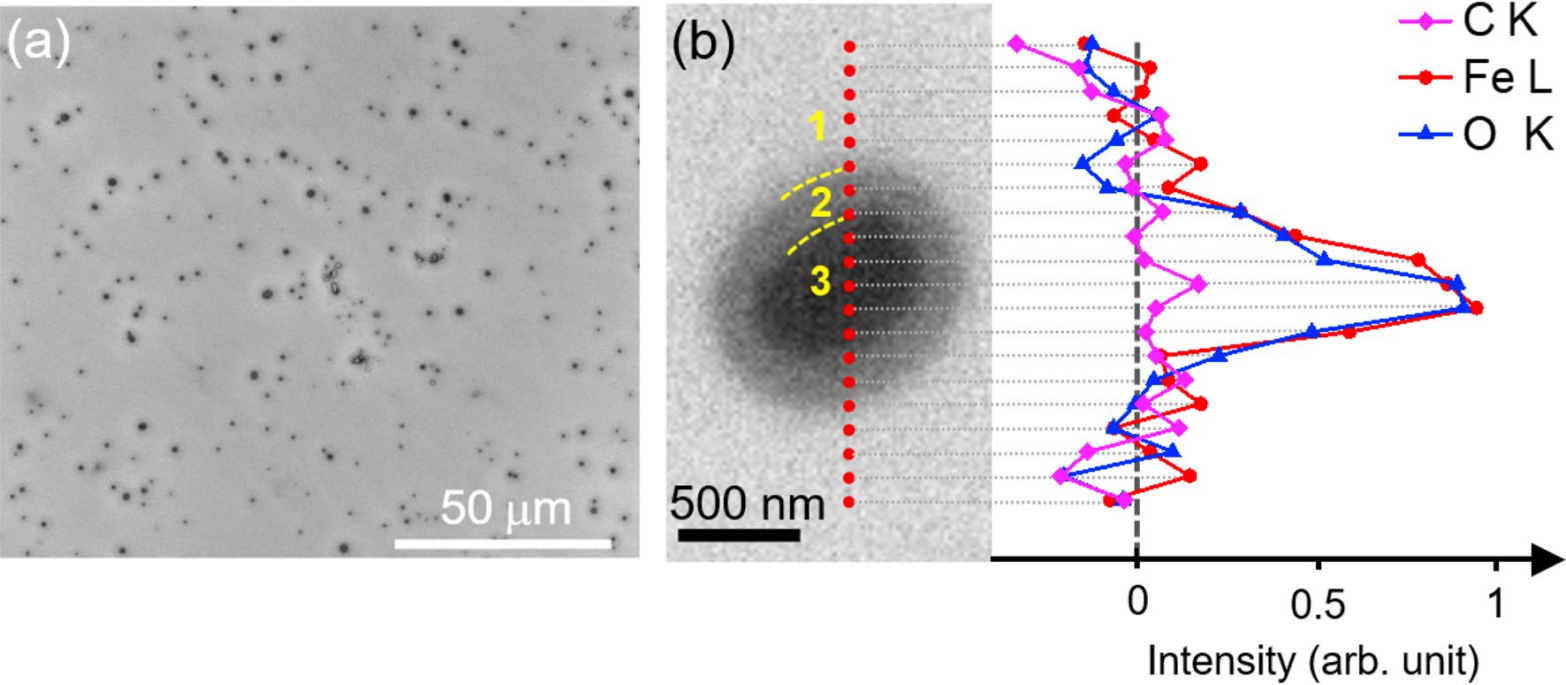
(**a**) SEM micrograph of the Fe_2_O_3_/GaN electrode surface. The dark spots are the Fe_2_O_3_ particles deposited as cocatalysts. The energy dispersive X-ray spectroscopy analysis along the red dotted line in (**b**) confirmed the presence of Fe and O only in the region 3.

We measured the size of the iron oxide particles using SEM observations. The particles had an average size of 1 μm. The optimal GaN surface coverage by the iron oxide particles was 1.3%, which agrees with previous reports of optimal GaN coverage by NiO cocatalyst^12^. Figure 1a shows the iron oxide particles deposited on GaN as dark spots. A magnification of one of those spots is shown in Fig. 1b, where three different-contrast regions are identified by numbers from 1 to 3. The Energy Dispersive X-ray spectroscopy (EDX) profiles of C, Fe and O along the red dotted line of Fig. 1b confirmed the presence of Fe and O only in region 3, and we discarded the non-uniform presence of carbon in the whole mapped region because the EDX carbon profile did not present significant variations. Therefore, the observation of the transition region (region 2) surrounding the Fe_2_O_3_ particle was not caused by different material compositions, though it can be related to a local change in the carrier concentration of GaN^33,34^.

The local variation in carrier concentration around the Fe_2_O_3_ particles can be originated either from the solid phase diffusion of Fe atoms into GaN during the annealing process after the cocatalyst deposition, or by the formation of a junction between GaN and Fe_2_O_3_. Although it has been shown that Fe atoms can diffuse into GaN as far as 1 μm if it is annealed at 1,050 °C^35^, the low temperature of our annealing process (500 °C) and the exponential dependence of the diffusion coefficient on temperature suggest that the doping effect is not considerable. Besides, Fe atoms in GaN act as acceptor-like point defects^36^, decreasing the n-type carrier concentration of GaN. Thus, a GaN region containing Fe diffused atoms would generate a brighter contrast in SEM than the unintentionally doped (uid)-GaN, rather than the darker contrast observed. Because Fe_2_O_3_ is typically an n-type semiconductor^37^, we believe that the formation of a n^–^-GaN/n-Fe_2_O_3_ junction is the cause of the transition region observed around the Fe_2_O_3_ particle. This heterojunction would induce a negative charge accumulation in the GaN region surrounding the Fe_2_O_3_ particle that should present a darker contrast in SEM than uid-GaN, which agrees with our observations.

**Figure 2:**
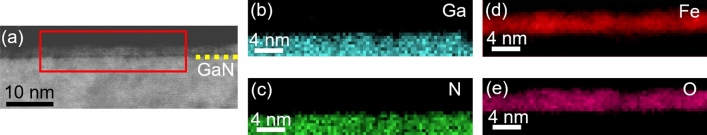
(**a**) Cross-sectional scanning TEM micrograph of the Fe_2_O_3_/GaN electrode. (**b**)–(**e**) EELS elemental mapping of Ga, N, Fe and O in the zone enclosed by the red rectangle marked in (**a**).

The identification of the iron oxide stoichiometry and phase were done by the study of the chemical and crystalline properties of the iron oxide particles. Figure 2a shows the scanning TEM micrograph of the interface between GaN and Fe_2_O_3_. Figure 2b–e show the EELS elemental mappings of Ga, N, Fe and O, respectively, of the area enclosed by the red rectangle in Fig. 2a. In those mappings, GaN and iron oxide regions are clearly separated, and there was no observable diffusion of O and Fe into the GaN region within the detection limits of the EELS analysis. The thickness of the iron oxide in the region observed was about 3 nm.

**Figure 3:**
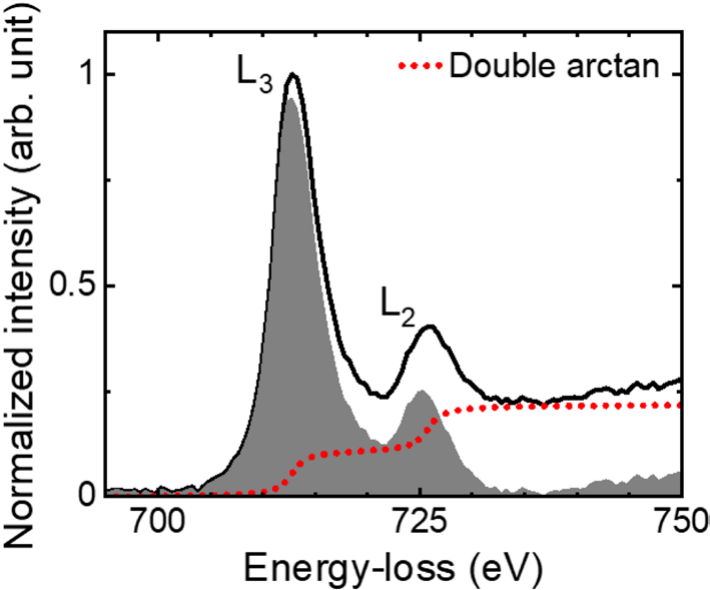
Electron energy-loss near-edge structure of Fe L_2,3_-edge, obtained from the iron oxide region analysed by EELS. The gray region represents the difference between the energy-loss spectrum (solid line) and the double arctan continuum model (broken red line).

The presence of the α phase of Fe_2_O_3_ can be confirmed by the ferric iron concentration (Fe^3+^/ΣFe) derived from the Fe electron Energy-Loss Near-Edge Structure (ELNES) presented in Fig. 3. The ELNES data was obtained from the iron oxide region mapped by EELS in Fig. 2. The maximum of the L_3_ and L_2_ peaks were found at 712.8 and 725.8 eV, respectively. The L_3_/L_2_ white-line intensity ratio and Fe^3+^/ΣFe were calculated after extracting the background with a double arctan function as the continuum model and using integration windows (2 eV width) centered on the peaks^38^. The L_3_/L_2_ white-line intensity ratio found in our analysis was 3.9, corresponding to a Fe^3+^/ΣFe of 44% based on the calibration done in ref.^38^, which is lower than the expected value of 100% in α-Fe_2_O_3_. Nonetheless, the iron in the cocatalyst particles was susceptible to the interaction with the carbon deposited directly on the photoelectrode surface by the electron beam during the lamella preparation. Therefore, given the thickness of the Fe_2_O_3_ particle, we believe that there was a substantial reduction of Fe^3+^ atoms to Fe^2+^ in the lamella preparation process.

**Figure 4:**
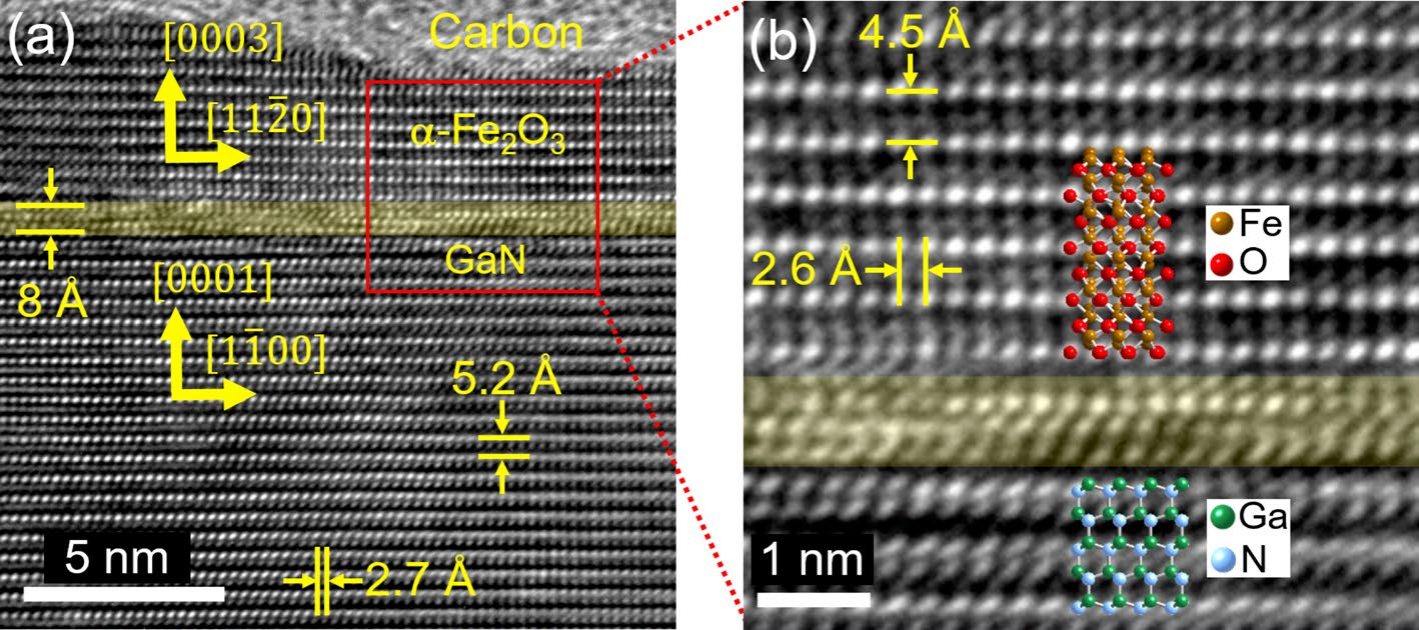
(**a**) HRTEM of the Fe_2_O_3_/GaN interface. (**b**) Magnification of the red rectangle highlighted in (**a**). Some crystallographic directions are displayed based on the lattice spacings of GaN and α-Fe_2_O_3_. In (**b**), the crystal projections of Fe_2_O_3_ and GaN oriented as indicated in (**a**) are overlapped in the lattice fringes in one of their possible configurations. The translucent yellow stripes in (**a**) and (**b**) show the zone of high distortion of lattice fringes at the Fe_2_O_3_/GaN interface.

Despite the Fe^3+^/ΣFe found was low to confirm that the iron oxide was α-Fe_2_O_3_, the lattice spacings observed through High-Resolution TEM (HRTEM) correspond only to α-Fe_2_O_3_ in the $[ {1{\bar{ 1}}00} ]$. zone axis and not to any other phase of Fe_2_O_3_ nor mixed oxide of Fe^2+^ and Fe^3+^. The HRTEM micrograph displayed in Fig. 4a shows the cross-sectional view of the Fe_2_O_3_/GaN interface. Based on the lattice spacings found and the lattice constants of α-Fe_2_O_3_ (*a* = 5.038 Å and *c* = 13.772 Å)^39^ and wurtzite GaN (*a* = 3.189 Å and *c* = 5.185 Å)^5^, some crystallographic orientations were marked in the Fe_2_O_3_ and GaN regions. The in-plane lattice mismatch between Fe_2_O_3_ and GaN is 58%. However, due to the orientation relationship between Fe_2_O_3_ and GaN, the interplanar spacing mismatch is only 9%.

Figure 4b shows a close-up of the red rectangle marked in Fig. 4a, with the GaN and Fe_2_O_3_ crystal projections superposed (oriented as indicated in Fig. 4a) in one of their possible configurations. The observation of high distortion of the lattice grids of GaN and Fe_2_O_3_ near their interface is localized in a transition layer with a thickness of only 8 Å, highlighted in yellow in Fig. 4a,b. The size of the transition layer and the evidence of no diffusion of iron into the GaN region indicates an abrupt Fe_2_O_3_/GaN interface. Even though we did not observe any constraint in the lattice size of Fe_2_O_3_ imposed by the influence of GaN, we recognized that the recrystallization orientation of Fe_2_O_3_ kept a particular epitaxial relationship, which followed the Fe_2_O_3_$\{ {0003}\}||$GaN$\{ {0001} \}$ and Fe_2_O_3_$[ {11{\bar{ 2}}0} ]||$GaN$[ {1{\bar {1}}00} ]$ symmetry constraints, equivalent to those previously reported^40^. Such epitaxial relationship is potentially generated due to the similar hexagonal symmetry of the planes perpendicular to the *c*-axis formed by oxygen in Fe_2_O_3_ and Ga or N in GaN.

**Figure 5:**
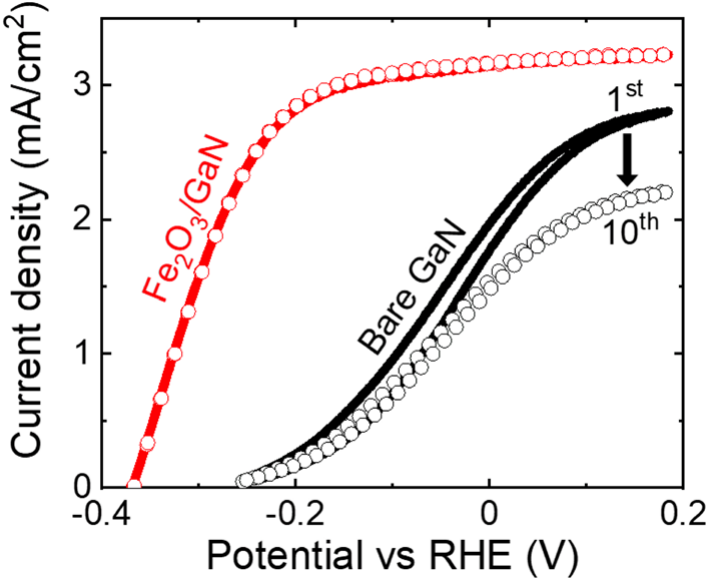
Cyclic voltammogram traces of the Fe_2_O_3_/GaN and bare GaN electrodes at a scanning speed of 20 mV/s. The solid lines represent the current density during the 1st cycle of the measurements and the scattered data during the 10th cycle.

Regarding the PEC characterization of the Fe_2_O_3_/GaN and bare GaN electrodes, the study of the oxidation half-reaction by Cyclic Voltammetry (CV) allowed us to compare qualitatively their performance as photoanodes. Figure 5 contains the first and tenth cycles of the CV measurements of such electrodes. The dark currents were negligible and are not shown. The election of measuring only 10 cycles in the CV characterization was done based on the quick degradation of the photocurrent of the bare GaN electrode, though the traces of the Fe_2_O_3_/GaN electrode were almost identical. The behavior of both electrodes in this characterization was similar but with a potential shifting: they presented a steady current density increase until they reach a plateau. The plateau level of the bare GaN electrode is barely reached within the voltage interval used in the measurements, but it is clearly reached in the Fe_2_O_3_/GaN electrode because of its large negative potential shifting. For example, at a current density of 1 mA/cm^2^, the potential of the Fe_2_O_3_/GaN electrode was about 0.2 V lower than the potential of the bare GaN electrode.

In general, the flat-band potential follows the trend of the onset voltage^41^. Thus, the observable shifting in the CV behavior of both electrodes can be attributed to the different potential at their surfaces, caused by the presence or absence of the cocatalyst. The negative potential shift and the higher photocurrent in the plateau region observed in the voltammogram of the Fe_2_O_3_/GaN electrode are evidence of its higher reaction rate compared with the bare GaN electrode^26^.

**Figure 6:**
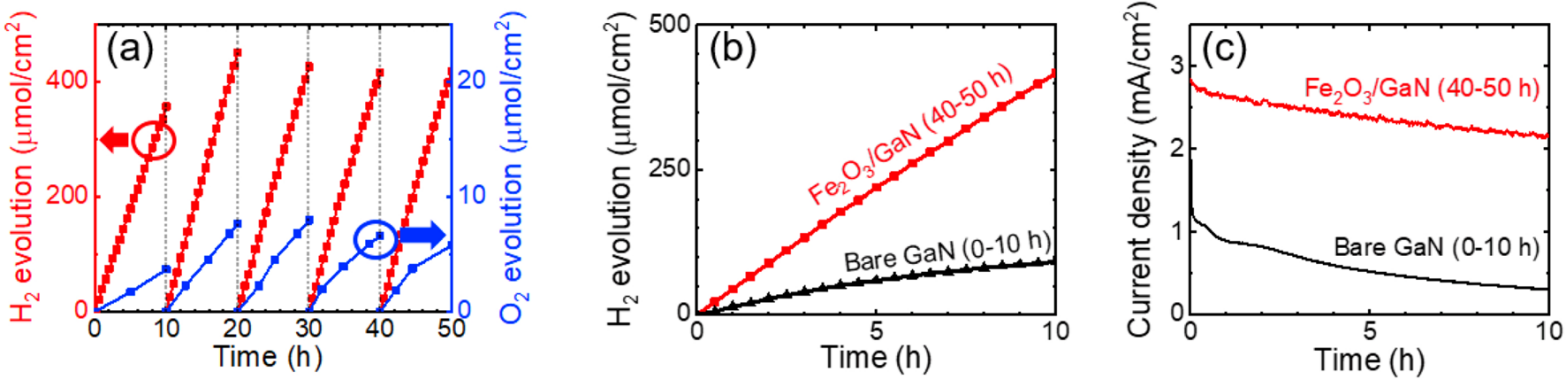
(**a**) H_2_ and O_2_ evolution on the platinum cathode and Fe_2_O_3_/GaN anode, respectively, during 50 h of zero-bias PEC characterization. Comparison of the (**b**) H_2_ evolution at the cathode and (**c**) photocurrent of the PEC cell during the fifth round of characterization (40–50 h) of the Fe_2_O_3_/GaN anode, and the unique characterization of the bare GaN anode (0–10 h).

The results of the two-electrode PEC characterization to study the zero-bias H_2_ generation of both photoelectrodes are shown in Fig. 6. The H_2_ and O_2_ generated by the platinum cathode and the Fe_2_O_3_/GaN anode, respectively, are presented in Fig. 6a, where it is shown that the H_2_ generation rate remained stable in each characterization, with an average over the five rounds of characterization of 11.5 nmol/cm^2^ s. As a comparison, Fig. 6b,c show the H_2_ generation and current density, respectively, of the PEC cell during the fifth round of characterization (40–50 h) of the Fe_2_O_3_/GaN anode, and the first and unique round of characterization of the bare GaN electrode (0–10 h). To compare the performance of both electrodes in this characterization, we used as a figure of merit the ECE, defined by:

1 $${\eta _H}=\frac{{n{{\Delta }}G}}{P}$$

where *n* is the H_2_ generation rate (mol/cm^2^ s), ${{\Delta }}G$ is the Gibbs free energy of H_2_ combustion with O_2_ (237.1 kJ/mol) and *P* is the optical power density irradiated (100 mW/cm^2^). The ECE of the of the Fe_2_O_3_/GaN electrode in its five rounds of characterization were 2.3%, 3.0%, 2.8%, 2.7% and 2.7%. The bare GaN electrode reached an ECE of only 0.6%. Based on the comparison of the light source used in our PEC experiments with the standard AM 1.5G spectrum, we can estimate that our calculated ECE is about 11 times higher than the standard solar-to-hydrogen ECE. The faradaic efficiencies of the H_2_ evolution in the five rounds of the PEC characterization of the Fe_2_O_3_/GaN electrode were 85%, 100%, 93%, 84% and 90%. Although the faradaic efficiencies related to the H_2_ generation agree with the values expected in water splitting, the O_2_ generation at the Fe_2_O_3_/GaN electrode was lower than the one expected in this reaction. The maximum H_2_:O_2_ ratio obtained was 2:0.03, indicating an oxygen generation of only 3% of the stoichiometric amount expected in water splitting. This result suggests that the photogenerated holes were not only involved in the O_2_ generation reaction, but also in other side reactions. For instance, Ito et al*.* (1984)^42^ demonstrated that the presence of epoxy resin in a PEC cell generate liquid organic byproducts when it is irradiated with UV light. Given that the illumination source of our experiments is mostly UV light, the decomposition of epoxy resin and the interaction of its organic byproducts and the photogenerated holes in the GaN anode can occur. In fact, the generation of bubbles at the anodes was poor, which indicates that the photoelectrodes were driving reactions whose byproducts were not in gas phase but in liquid phase. Those reactions may have a lower potential than the necessary for water splitting, therefore, the ECE parameter calculated as a figure of merit by Eq. (1) would be overestimated compared with the real ECE of the electrodes.

**Figure 7:**
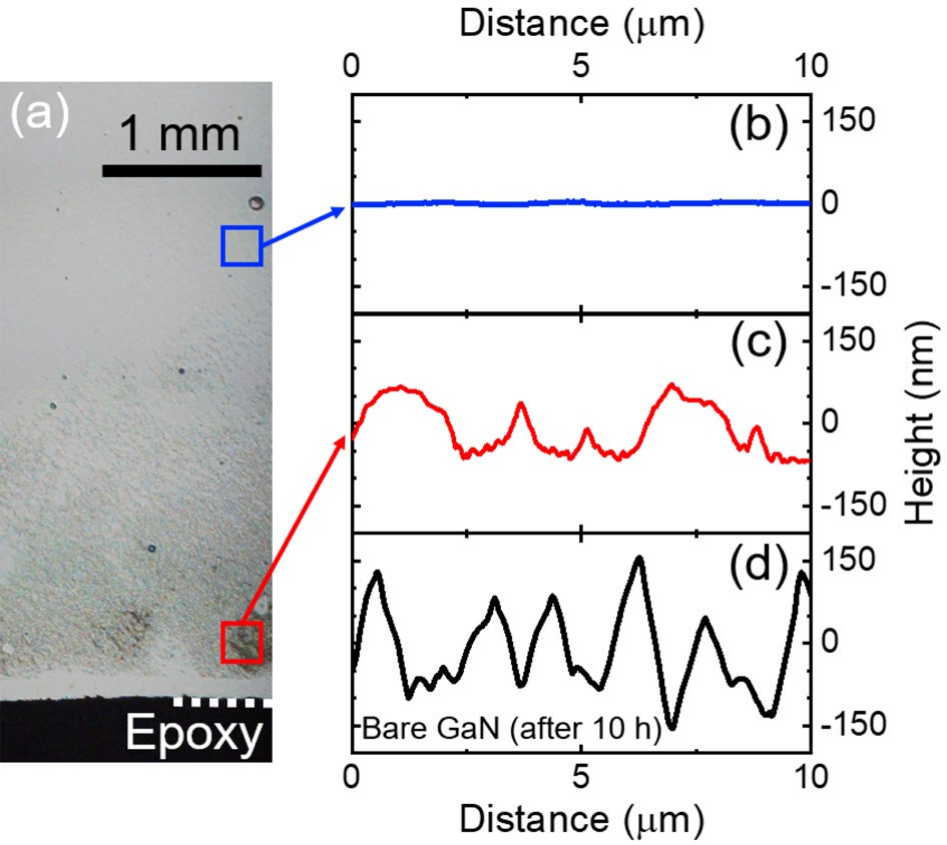
(**a**) Optical microscope image of the surface of the Fe_2_O_3_/GaN electrode after 50 h of PEC characterization. At the bottom, the epoxy resin covering the metallic contact of the electrode is observable. The AFM topographic profiles of the Fe_2_O_3_/GaN electrode around the blue and red regions marked in (**a**) are presented in (**b**) and (**c**), respectively. The topographic profile of the bare GaN photoelectrode surface after 10 h of PEC characterization is presented in (**d**).

One of the benefits of incorporating a cocatalyst in the GaN electrode surface is the prevention or retardation of the GaN photocorrosion. While the bare GaN electrode exhibited macroscopic signals of photocorrosion^43,44^ within the first 2 h of PEC characterization (loss of the mirror-like finish of the GaN surface), the Fe_2_O_3_/GaN electrode exhibited these photocorrosion marks after 43 h and only near the metallic contact of the electrode. Thus, the appearance of photocorrosion marks was retarded more than 20 times. Figure 7a shows a picture of the surface of the Fe_2_O_3_/GaN electrode after 50 h of two-electrode PEC characterization, where the metallic contact region covered with epoxy resin is observable at the bottom of the picture. The Atomic Force Microscope (AFM) topographic profile in Fig. 7b of the electrode a few millimeters away from the metallic contact (blue square in Fig. 7a) shows that the electrode preserves its smooth flat surface in that region. In contrast, the topographic profile presented in Fig. 7c of the region close to the metallic contact (red square in Fig. 7a) shows an etching depth of at least 140 nm. As a comparison, the topographic profile of the bare GaN electrode after the two-electrode PEC characterization is shown in Fig. 7d. This electrode exhibited etched regions with a depth of more than 300 nm; however, the etching was present over the whole electrode surface and only after 10 h of characterization. The localized surface damage of the Fe_2_O_3_/GaN can be explained by the short distance between its metallic contact and the etched regions. The electron extraction is more difficult in the region far from the contact than in the region close to it due to the higher electrical resistance that the n-GaN layer represents. Therefore, the recombination of the electron–hole pairs generated during the photoabsorption process will be higher in the region far from the contact. The higher population of free holes near the contact would be able to drive the oxidation of species more easily, including GaN, which would be reflected in a faster photocorrosion of the electrode in that region^45^.

The H_2_ generation of our PEC cell using the Fe_2_O_3_/GaN and bare GaN electrodes is consistent with their CV characterizations. The best performance observed in both PEC characterizations corresponds to the Fe_2_O_3_/GaN electrode. As mentioned above, the bare GaN electrode suffered a quick degradation in their photocurrent, observable in the difference of the photocurrent trace in as few as ten cycles of its CV characterization, and also observable in the quick decay of its photocurrent in the first hour of its two-electrode characterization, presented in Fig. 6c. Despite the Fe_2_O_3_/GaN electrode has demonstrated an improvement in the photocatalytic reaction and photocorrosion retardation, its performance is slightly inferior when compared with a NiO/GaN electrode. Under the same experimental conditions, a NiO/GaN electrode reached a maximum ECE of 3.3% (see Supplementary Figure S1), and after 50 h of PEC characterization it did not show any marks of photocorrosion. The O_2_ generated by this electrode was 11% of the amount expected in water splitting, and while it is still low, it is certainly higher than the 3% generated by the Fe_2_O_3_/GaN electrode.

The improvement of the catalytic activity of the Fe_2_O_3_/GaN electrode due to the presence of the iron oxide particles might be a consequence of the introduction of active sites either by the Fe or O atoms. However, if this is the case, an Fe_2_O_3_ thin film would improve further the catalytic activity of GaN due to a larger introduction of active sites. To verify this idea, we prepared and characterized an Fe_2_O_3_ (thin film)/GaN electrode. The thickness of the Fe_2_O_3_ layer was 2.5 nm (see Supplementary Figure S2), comparable with the thickness of the iron oxide particles (3 nm) and thus presenting a similar electrical resistance between GaN and the electrolyte. The Fe_2_O_3_ layer also exhibited and epitaxial relationship with GaN, but with the Fe_2_O_3_$\{{11{\bar {2}}0}\}||$GaN$\{{0002}\}$ and Fe_2_O_3_$[ {3{\bar{ 3}}00} ]||$GaN$[ {11{\bar{ 2}}0} ]$ symmetry constraints (see Supplementary Figure S3), different from the constraints found in the Fe_2_O_3_ particles. The ECE of this electrode reached a value of only 0.6% (see Supplementary Figure S4), which is the same value reached by the bare GaN electrode, but after 10 h of PEC characterization it did not show any marks of photocorrosion. The inferior catalytic performance of a GaN electrode when using an Fe_2_O_3_ thin film as cocatalyst instead of Fe_2_O_3_ particles can be caused by the different crystallographic planes at the surface of the Fe_2_O_3_ given the different epitaxial relations found. Nonetheless, the catalytic properties of the Fe_2_O_3_$( {11{\bar{ 2}}0} )$ and Fe_2_O_3_$( {0001})$ orientations seem to be similar. For example, theoretical calculations have shown that the OER overpotential between the Fe_2_O_3_$( {11{\bar{ 2}}0} )$ and Fe_2_O_3_$( {0001} )$ orientations is not significant^46^. Thus, the performance of the Fe_2_O_3_ (thin film)/GaN electrode is inferior because the active sites are not introduced by the iron oxide. Instead, we believe that the improvement of the catalytic activity might be a consequence of the formation of accumulation layers in GaN (region 2 in Fig. 1b) due to the n^–^-GaN/n-Fe_2_O_3_ junction. The extraction of holes in the junction between these accumulation layer regions and the electrolyte would be improved by the higher band bending of GaN at the surface of these regions compared with the original uid-GaN (as a result of their difference in carrier concentration). The sparsity of such regions also brings the advantage of preserving the photoabsorption properties of the bulk GaN.

## Methods

### Photoelectrode structure

**Figure 8:**
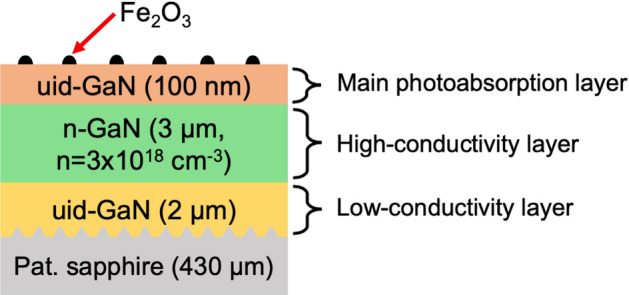
Schematic of the cross-sectional view of the Fe_2_O_3_/GaN electrode.

The GaN structure of the photoelectrodes was grown by metalorganic vapor phase epitaxy on a *c*-plane conical-patterned sapphire substrate (PSS) with a pattern pitch, diameter, and height of 3.0, 2.6 and 1.6 μm, respectively. The homostructure consisted of (top to bottom) uid-GaN (100 nm)/n-GaN (Si-doped, n = 3 × 10^18^ cm^−3^, 3 µm)/uid-GaN (2 µm). A schematic of the cross-sectional view of the Fe_2_O_3_/GaN electrode is presented in Fig. 8. The PSS improves the crystalline quality of the electrode structure because when GaN is grown on it, its dislocations tend to merge, reducing the overall number of crystal defects. Given that the initial GaN layer has the possibility of trapping carriers via defects, it needs to be non-conductive. Therefore, the underlying GaN was chosen to be unintentionally doped so it has a low electrical conductivity. The objective of the top uid-GaN layer was to extend the depletion layer to a thickness comparable to the light penetration depth of GaN. This increase of the depletion layer optimize the charge separation after the photoabsorption process^47^. While the holes are directed to the surface of the electrode to perform oxidation reactions, the electrons should be transported out of the GaN structure to the counterelectrode, where the reduction reactions take place. However, uid-GaN has a high electrical resistance, thus, to facilitate the electron extraction of the GaN photoelectrode, we introduced the underneath n-GaN layer, which provides a low-resistance electrical path.

### Cocatalyst deposition

The deposition of the Fe_2_O_3_ and NiO cocatalysts was done by spin coating a diluted Metalorganic Decomposition Solution (MOD). The MOD solution contained 3 wt% of either Fe_2_O_3_ or NiO. The MOD solution for the Fe_2_O_3_ particles deposition was diluted in a Fe_2_O_3_ MOD:butyl acetate:H_2_O ratio (by volume) of 2:100:30, and for the Fe_2_O_3_ thin film it was diluted in a Fe_2_O_3_ MOD:butyl acetate ratio of 1:20. In the case of the NiO cocatalyst, the NiO MOD was diluted in a NiO MOD:butyl acetate:H_2_O ratio of 11:1,000:300. All the substrates were spun at a speed of 2,000 rpm for 30 s after the cocatalyst solution was deposited on each one and, ultimately, annealed at 500 °C for 30 min in air.

### Photoelectrode preparation

The substrates were prepared as photoelectrodes by soldering a copper wire to their surface using indium to create an ohmic contact between the wire and GaN. The copper and indium on the surface of the photoelectrode were electrically isolated by covering them with epoxy resin. In this manner, once the photoelectrodes were immersed in the aqueous solution (1 M NaOH) for the PEC characterizations, no metal was in contact with the electrolyte.

### Electron microscopy analyses

The surface of the Fe_2_O_3_/GaN electrode was characterized by SEM observations complemented with EDX using a FEI Magellan microscope operating at 3 and 5 kV. For the TEM analysis, a cross-sectional TEM lamella was prepared by focused ion beam (Ga) milling using a FEI Helios DualBeam (FIB/SEM) microscope. The surface of the lamella region was protected with electron-beam-deposited carbon and platinum, followed by ion-beam-deposited platinum. HRTEM observations and EELS analyses were done in a FEI Titan microscope operating at 300 kV. The EELS data was obtained in the scanning TEM mode using the dual-EELS technique to simultaneously acquire the core-loss and low-loss spectra. The energy dispersion was 0.5 V per channel and the energy drift was corrected by correlating the zero-loss peak positions obtained in the low-loss spectra with the core-loss spectra.

### PEC characterizations

The PEC characterizations of the photoelectrodes were done in two parts: linear sweeping CV in a three-electrode setup (sweeping speed of 20 mV/s) and measurements of the zero-bias H_2_ generation in a two-electrode setup. In the CV measurements, a reference electrode for alkaline solutions was used, which was calibrated with a Ag/AgCl/KCl (sat.) electrode. The potentials were converted to the RHE scale using the Nernst equation. In the two-electrode characterization, a platinum wire was utilized as a cathode, connected directly to the photoelectrode through an ammeter. In both PEC characterizations, the photoelectrodes were irradiated with a power density of 100 mW/cm^2^ using a 300 W Xe arc lamp filtered with a UV spectroscopic mirror^25^. The number of absorbable photons by GaN (wavelength < 362 nm) in our light irradiation setup was 3.8 × 10^16^ cm^−2^ s^−1^, which is about 11 times the number of absorbable photons in the AM 1.5G solar spectrum. The two-electrode characterizations were done in rounds of 10 h each, and during these characterizations, the amount of the gases generated on the electrodes was recorded and they were characterized later using gas chromatography. The faradaic efficiencies of the H_2_ generation were calculated using the next equation

2 $${\eta _F}=\frac{{{\eta _H}P}}{{\left( {1.23\,V} \right)\left( {\bar {J}} \right)}}$$

where ${\eta _H}$ is the H_2_ ECE described by Eq. (1), $P$ is the irradiation power density (100 mW/cm^2^), ${\bar{ J}}$ is the average current density of the photoelectrode and 1.23 *V* represent the difference in formal potentials of the hydrogen-evolution and oxygen-evolution half-reactions.

### AFM profiles

The topographic profiles of the GaN electrodes after their PEC characterizations were measured with an Agilent 5500 Scanning Probe Microscope with a Bruker RTESPA-300 probe.

## Conclusions

The PEC characterizations performed showed an improvement of the catalytic activity in a GaN photoelectrode when it is covered by Fe_2_O_3_ particles as cocatalyst. We found that the H_2_ generation rate is about 5 times higher in a Fe_2_O_3_/GaN electrode compared with a bare GaN electrode. Besides, while the photocorrosion in the bare GaN electrode was macroscopically observed after just 2 h of PEC characterization, some minor photocorrosion damage was observed in the Fe_2_O_3_/GaN electrode only after 43 h.

In the study of the crystalline properties of the cocatalyst particles, we identified that they keep an epitaxial relationship with GaN, which follows the Fe_2_O_3_$\{ {0003} \}||$GaN$\{ {0001}\}$ and Fe_2_O_3_$[{11{\bar {2}}0}]||$GaN$[ {1{\bar {1}}00}]$ symmetry constraints. When the Fe_2_O_3_ was deposited as a thin film, we observed a different epitaxial relationship, with the Fe_2_O_3_$\{ {11{\bar{ 2}}0} \}||$GaN$\{ {0002}\}$ and Fe_2_O_3_$[ {3{\bar{ 300}}} ]||$GaN$[ {11{\bar {20}}} ]$ symmetry constraints. However, the thin film cocatalyst did not show any improvement of the catalytic properties of GaN.

The incorporation of Fe_2_O_3_ onto the surface of a GaN photoelectrode improved its chemical endurance as well as its photocatalytic activity. Thus, Fe_2_O_3_ proved to be a suitable cocatalyst of GaN, which expands the technological applications possibilities of nitride-based semiconductors.

## Supplementary information

Supplementary Information.

## Data Availability

All data generated or analysed during this study are included within the article (and its supplementary information) or are available from the corresponding author upon reasonable request.
