# Supplementary material for: Photoelectrochemical and crystalline properties of a GaN photoelectrode loaded with α-Fe2O3 as cocatalyst
Source: Sci Rep. 2020 Jul 28;10:12586. doi: 10.1038/s41598-020-69419-8 (PMC7387544; doi:10.1038/s41598-020-69419-8)
Supplement: Supplementary file 1 — Supplementary Information. [file 41598_2020_69419_MOESM1_ESM.pdf]

Photoelectrochemical and Crystalline Properties of a GaN Photoelectrode Loaded with α-Fe_2O_3 as Cocatalyst

Martin Velazquez-Rizo, Daisuke Iida, and Kazuhiro Ohkawa*

Computer, Electrical and Mathematical Sciences and Engineering (CEMSE) Division, King Abdullah University of Science and Technology (KAUST), Thuwal 23955-6900, Saudi Arabia.

*Corresponding author: kazuhiro.ohkawa@kaust.edu.sa

Supplementary data

PEC characterization of the NiO/GaN electrode.

See the section *Methods* for the experimental conditions of the NiO deposition and two-electrode PEC characterization.

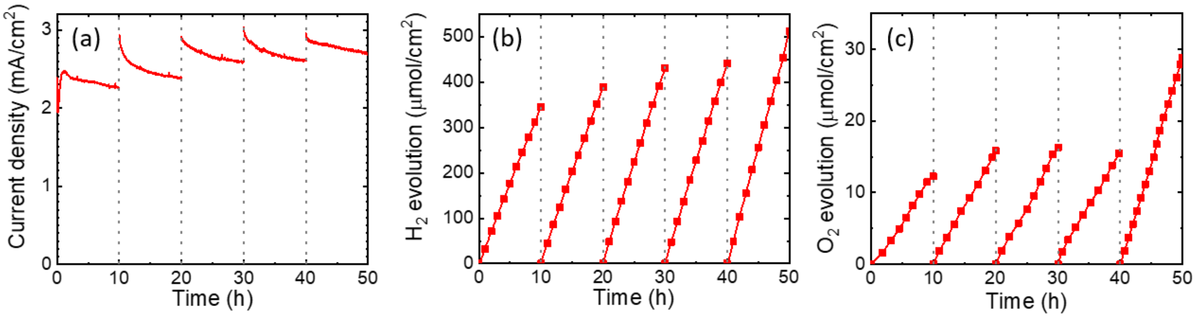

Supplementary Figure S1. (a) Current density, (b) H_2 evolution and (c) O_2 evolution of the NiO/GaN electrode during its two-electrode PEC characterization.

The energy conversion efficiencies (see eqn. 1 in the section *Results and Discussion*) of the NiO/GaN electrode in its 5 PEC characterizations rounds were 2.2%, 2.5%, 2.8%, 2.8% and 3.3%. The H_2 generation faradaic efficiencies (see eqn. 2 in the section *Methods*) in those characterizations were 76%, 81%, 85%, 85% and 97%. The largest amount of O_2 generated during one round of PEC characterization was 11% of the stoichiometric amount expected in water splitting.

Characterization of the Fe_2O_3 (thin film)/GaN electrode.

See the section *Methods* for the experimental conditions of the Fe_2O_3 thin film deposition and two-electrode PEC characterization. The thickness and crystallographic structure were characterized using TEM and scanning TEM images.

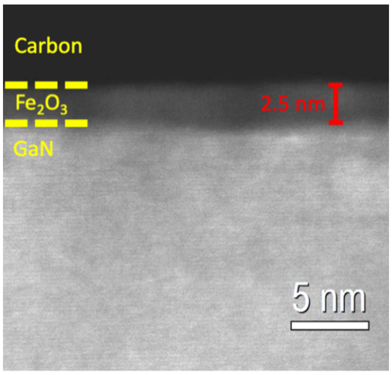

Supplementary Figure S2. Cross-sectional scanning TEM micrograph of the Fe_2O_3 (thin film)/GaN electrode. The thickness of the Fe_2O_3 thin film is 2.5 nm.

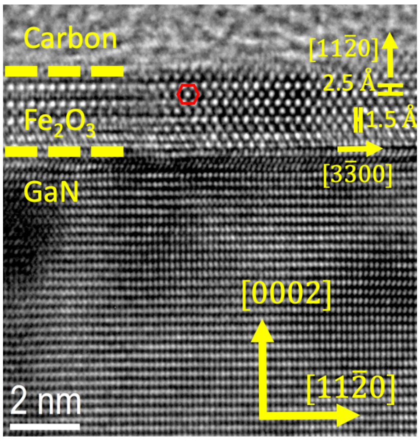

Supplementary Figure S3. Cross-sectional HRTEM micrograph of the Fe_2O_3 (thin film)/GaN electrode. Two crystallographic directions and interplanar spacings of the Fe_2O_3 thin film and two crystallographic directions of GaN are indicated. The red hexagon marked in the Fe_2O_3 area matches the hexagonal symmetry of the Fe-atoms columns present along the $Fe_2O_3[0001]$ zone axis.

The scanning TEM micrograph shown in Supplementary Figure S2 allowed to measure the thickness of the iron oxide film based on the contrast between Fe_2O_3 and GaN. The thickness of the oxide film was about 2.5 nm. Based on the HRTEM micrograph displayed in Supplementary Figure S3, we identified different crystallographic orientations of Fe_2O_3 and GaN. In this case, we also observed an epitaxial relationship between Fe_2O_3 and GaN with the $Fe_2O_3\{11\bar{2}0\}||GaN\{0002\}$ and $Fe_2O_3[3\bar{3}00]||GaN[11\bar{2}0]$ symmetry constraints, different from the ones observed in the iron oxide particles deposited on GaN.

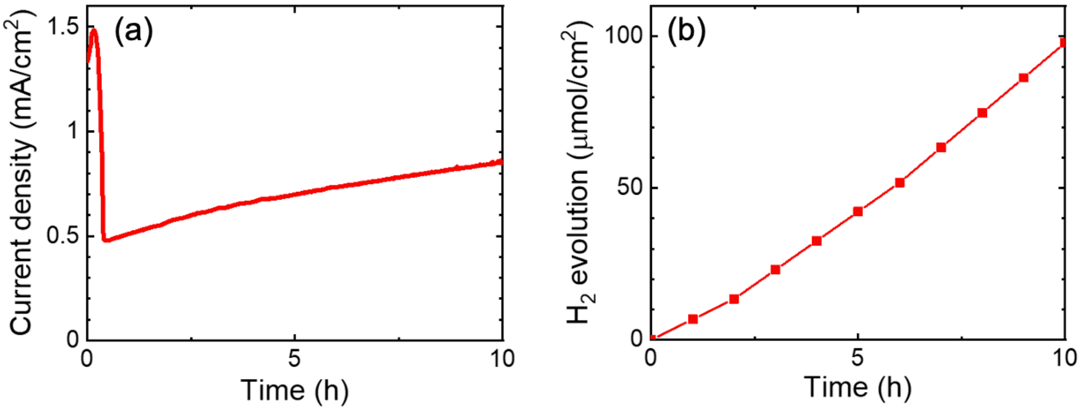

Supplementary Figure S4. (a) Current density and (b) H_2 evolution of the Fe_2O_3 (thin film)/GaN electrode during its two-electrode PEC characterization.

The energy conversion efficiency and the H_2 generation faradaic efficiency (see eqn. 1 in the section *Results and Discussion* and eqn. 2 in the section *Methods)* of the Fe_2O_3 (thin film)/GaN electrode were 0.63% and 0.72%, respectively. The O_2 generation on this electrode was only 2 µmol at the end of its 10 h PEC characterization.
